# Aberrant Thyroid Function Tests in a Patient Taking Biotin Supplements

**DOI:** 10.7759/cureus.5297

**Published:** 2019-08-01

**Authors:** Zara Latif, Marc J Crupie

**Affiliations:** 1 Internal Medicine, University of Tennessee Health Science Center, Memphis, USA; 2 Family Medicine, University of Tennessee Health Sciences Center, Memphis, USA

**Keywords:** biotin, biotin-streptavidin immunoassay, thyroid, thyrotoxicosis, thyroid autoimmunity, thyroid dysfunction

## Abstract

Biotin is a component of the B-complex vitamins that has been widely used in over-the-counter supplements. The effects of biotin on thyroid function tests (TFTs) have been recently reported by multiple authors. We report here a case of a patient who presented with TFTs consistent with hyperthyroidism and a positive radioactive iodine uptake (RAIU) scan while taking biotin supplements. The TFTs normalized almost a month later and continued to be normal even after resumption of biotin supplements. Interpreting TFTs while patients are taking biotin could pose a diagnostic challenge, so we suggest more frequent monitoring for those patients before starting them on long-term anti-thyroid medications.

## Introduction

Biotin is a water-soluble vitamin that has been recently used in many over-the-counter supplements to improve hair and nail health [1]. The recommended daily dose of biotin is 30 μg/day [2]. It has been reported that biotin interferes with chemical assays that measure thyroid function tests (TFTs) [3]. Many assays use biotin streptavidin interaction due to biotin binding in high affinity to streptavidin [2]. This immobilization system produces false positive results on standardized thyroid panel laboratory values. Lab results showing thyrotoxicosis in asymptomatic patients taking biotin have been reported in multiple cases [4]. However, results of radioactive iodine uptake (RAIU) scan while a patient is taking biotin are not well reported.

The biotin streptavidin immunoassay is often used to measure serum levels of thyroid stimulating hormone (TSH), thyroid hormones, and thyroid antibodies. Two main assay methods are used to measure thyroid hormones, the “sandwich” assay and the competitive assay. Depending on the type of assay, biotin interference can produce a falsely elevated or depressed hormone levels. A “sandwich” assay is used to measure TSH. In a “sandwich” assay, a capture antibody that is conjugated with biotin binds streptavidin-coated microparticles. When there is high concentration of free biotin in the sample, the free biotin competes with the capture antibody for the binding site on the streptavidin molecule. This creates a low response signal and produces a falsely low TSH level when measured with that assay format [2, 5]. 

We report here a case of a woman on high dose biotin who presented with mildly symptomatic hyperthyroidism, markedly abnormal thyroid functions, and a positive RAIU scan. All the abnormal laboratory values resolved within a month and her TFTs remained normal after the resumption of biotin supplements. 

## Case presentation

A 34-year-old female with a medical history of anxiety and depression presented to her primary care physician due to increased anxiety and palpitations. She exhibited new complaints of muscle weakness, difficulty sleeping, and severe sweating for three months. Her medications included clonazepam 1 mg twice daily, sertraline 100 mg once daily, and biotin 20,000 μg once daily. She was taking biotin supplements sporadically for several months prior to her presentation. Vital signs during the visit were heart rate 70/min, blood pressure 126/74 mmHg, and respiration 12 breaths/min. Physical exam of thyroid gland revealed no goiter or enlargement. Initial labs included TSH which was suppressed at 0.19 mIU/L (normal 0.4-4.100 mIU/L) and free thyroxine (T4) was elevated at 2.08 ng/dL (normal 0.8-1.9 ng/dL). Thyroid peroxidase (TPO) antibodies were elevated at 147 IU/mL (normal 0-34 IU/mL) and total triiodothyronine (T3) was also elevated at 404.20 ng/dL (normal 80-200 ng/dL). Her thyroglobulin antibody (TgAb) was 50 IU/mL which was within normal limits (<115 IU/mL). Six days after labs were taken, thyroid uptake scan was performed demonstrating 24 h diffuse uptake of 53%, which was consistent with a diagnosis of Graves’ disease. Her primary care physician elected not to proceed with radioactive iodine or begin antithyroid medications at that time.

The patient was referred to endocrinology and evaluated three weeks later. She stopped taking her biotin and her labs normalized. Her TSH, free T4, free T3, and TPO levels all decreased (Table 1). A month after her visit to the endocrinologist, the patient resumed taking her biotin 20,000 μg daily and continued to be asymptomatic. A thyroid panel was repeated three months after she resumed biotin and her hormone levels remained within normal limits (Table 1). She remained asymptomatic with no signs of hyperthyroidism. 

**Table 1:** Patient’s clinical course and correlated laboratory values. TSH, thyroid stimulating hormone; T4, thyroxine; T3, triiodothyronine; Anti-TPO, thyroid peroxidase antibodies; TgAb, thyroglobulin antibody.

	4/17/2018	5/15/2018	10/15/2018	Reference range
Biotin use	On biotin supra-physiological doses	Off any biotin for 3 weeks	On biotin supra-physiological doses for 17 weeks	
TSH	0.19	2.6	3.26	0.4-4.1 mIU/L
Free T4	2.08	1.41	1.18	0.8-1.9 ng/dL
Total T3	404.2	-	101	80-200 ng/dL
Anti-TPO	147	12	-	0-34 IU/mL
TgAb	50	10	-	<115 IU/mL

## Discussion

This patient had transiently abnormal TFTs and positive radioiodine uptake. One explanation of these observed abnormal results is biotin interference with the chemical assay. She was taking supraphysiologic doses of biotin which could have influenced the results. Li et al. have demonstrated that biotin could interfere with the assay and result in suppressed TSH and elevated free T4, producing a false diagnosis of thyrotoxicosis [6]. This explanation, however, fails to consider the positive RAIU scan in this patient. To our knowledge, there are currently no reports on the interference of biotin with radioiodine uptake scans. Interestingly, our patient had mild symptoms consistent with hyperthyroidism while most patients with thyrotoxicosis due to biotin interference presented with minimal or no symptoms [4, 7, 8]. 

The normal TFT results after her resumption of biotin was perplexing due to her initial thyroid function abnormalities which were assumed to be biotin related. Ardabilygazir et al. report restarting their patient on biotin supplements after the initial TFT abnormalities were found but follow up TFTs were not reported [9]. We did not find reports of TFT results after biotin resumption in patients who previously had biotin interference. As our patient remained euthyroid on the same dose of biotin she was initially taking when the diagnosis was made, we concluded that her biotin intake does not fully explain her clinical course. 

The possibility of this patient having an autoimmune process unrelated to biotin supplements cannot be definitively excluded. The presence of elevated anti-TPO antibodies could support an autoimmune condition, however, anti-TPO antibodies has been reported in healthy adults with a prevalence up to 13% [10]. Her positive RAIU scan in the presence of little to no thyrotoxicosis symptoms was also puzzling. RAIU scans are positive in cases of hyperthyroidism due to Graves’ disease, iodine deficiency, or pregnancy [11]. As our patient did not fit any of these conditions, biotin remains the most likely culprit for the positive result through an unknown mechanism. 

## Conclusions

Thyroid function tests in patients taking biotin may show false results of thyrotoxicosis. In this case we demonstrated that in a symptomatic patient taking biotin, TFT and a positive RAIU scan could be extremely challenging to interpret. We suggest in a patient taking biotin, frequent follow up and monitoring of clinical symptoms as well as repeated laboratory follow up before starting treatment may be a beneficial approach. This approach can prevent subjecting patients to unnecessary procedures or potentially lifelong treatment for a presumptive diagnosis of Graves’ disease. 
